# Astragalus Polysaccharides and Metformin May Have Synergistic Effects on the Apoptosis and Ferroptosis of Lung Adenocarcinoma A549 Cells

**DOI:** 10.3390/cimb46080461

**Published:** 2024-07-23

**Authors:** I-Yun Lee, Ting-Chung Wang, Yu-Jen Kuo, Wei-Tai Shih, Pei-Rung Yang, Cheng-Ming Hsu, Yu-Shih Lin, Ren-Shyang Kuo, Ching-Yuan Wu

**Affiliations:** 1 Department of Chinese Medicine, Chiayi Chang Gung Memorial Hospital, Chiayi 61363, Taiwan; yunlee@livemail.tw (I.-Y.L.); ati8955@cgmh.org.tw (W.-T.S.); pamyang@cgmh.org.tw (P.-R.Y.); emptysecure99@gmail.com (R.-S.K.); 2 School of Chinese Medicine, College of Medicine, Chang Gung University, Taoyuan 33302, Taiwan; 3 Department of Neurosurgery, Chiayi Chang Gung Memorial Hospital, Chiayi 61363, Taiwan; northernblotting@gmail.com (T.-C.W.); ilucfe@hotmail.com (Y.-J.K.); 4 Department of Otolaryngology-Head and Neck Surgery, Chiayi Chang Gung Memorial Hospital, Chiayi 61363, Taiwan; scm00031@gmail.com; 5 School of Medicine, College of Medicine, Chang Gung University, Taoyuan 33302, Taiwan; 6 Cancer Center, Chiayi Chang Gung Memorial Hospital, Chiayi 61363, Taiwan; 7 Department of Pharmacy, Chiayi Chang Gung Memorial Hospital, Chiayi 61363, Taiwan; yohimba@cgmh.org.tw; 8 Institute of Molecular Biology, National Chung Cheng University, Chiayi 62102, Taiwan

**Keywords:** Astragalus polysaccharides (APS), metformin, apoptosis, ferroptosis, lung cancer, A549 cells

## Abstract

Astragalus polysaccharides (APSs), the compounds extracted from the common herb Astragalus membranaceus, have been extensively studied for their antitumor properties. In this study, we investigated the effect of APS on lung adenocarcinoma A549 cells. The effects of APS and the anti-diabetic drug metformin on apoptosis and ferroptosis were compared. Furthermore, the combination treatment of APS and metformin was also investigated. We found that APS not only reduced the growth of lung cancer cells but also had a synergistic effect with metformin on A549 cells. The study results showed that it may be promising to use APS and metformin as a combination therapy for the treatment of lung adenocarcinoma.

## 1. Introduction

Lung adenocarcinoma usually evolves from the mucosal glands and represents about 40% of all lung cancers [1]. However, as the treatment progresses, the 5-year survival is less than 12% to 15%. Lung adenocarcinoma has become the most prevalent non-small cell lung cancer in the last two decades [2]. It is also the most common primary cancer in the United States.

The standard treatment of lung adenocarcinoma includes surgery, radiation, and chemotherapy. However, the adverse effects may be so severe that patients’ treatment plan would be discontinued [3]. Therefore, new antitumor agents with fewer side effects are always expected.

Traditional Chinese medicine (TCM) has been widely used in Taiwan, China, Japan, and South Korea for a long time [4]. There are numerous studies on using TCM as adjuvant therapy for treating cancer. Its ability to enhance efficacy and reduce side effects during either chemotherapy or radiotherapy has been reported [5]. It has also been reported that, for lung cancer patients, TCM may serve as a possible adjunctive treatment [6].

*Astragalus membranaceus* is among the most frequently prescribed TCM herbs for lung cancer patients according to the analyses of the Taiwan National Health Insurance Database [6,7,8]. *A. membranaceus* is a prevalent herb that is used not only as a remedy but also for food consumption. In traditional Chinese medicine theory, the effect of *A. membranaceus* is to invigorate the spleen and replenish qi. It is widely used in Asian countries [9].

The Astragalus polysaccharide (APS) extracted from *A. membranaceus* is a type of bioactive water-soluble heteropolysaccharide. APS components differ from the plant varieties, origins, and extraction methods. Additionally, their biological activities are affected by their chemical structures [10] and monosaccharide compositions [11]. The structures are commonly measured with the following tools: high-performance liquid chromatography (HPLC), gas chromatography (GC), mass spectrometry (MS), and nuclear magnetic resonance (NMR) [11,12].

It is hard to determine an accurate composition of APS due to its complicated chemical structure. The monosaccharide composition and proportion of polysaccharides with varying molecular weights are distinct. Consequently, the sugar chain connection sequence, glycosidic bond types, and corresponding biological activities exhibit differences [9].

One study investigated 24 types of polysaccharides extracted from the root of *A. membranaceus*, the majority of which were heteropolysaccharides. The molecular weight of heteropolysaccharides ranges from 8.7 to 4800 kDa. They are composed of different monosaccharides, including L-rhamnose, L-rabinose, D-xylose, L-xylose, D-ribose, L-ribose, D-galactose, D-glucose, and D-mannose [13].

APS has been reported to be effective in various aspects, such as anti-diabetic [14], antitumor [15], immunomodulation [16], etc. For cancer treatment, APS has the following effects: inhibiting the proliferation of tumor cells [17,18], inducing the apoptosis of tumor cells [19,20,21], and inhibiting the transfer of tumor cells [22,23].

As an adjuvant therapy, APS in combination with adriamycin was reported to promote the apoptotic effect of the chemotherapy adriamycin in gastric cancer cells [24] and enhance the antitumor effect in H22 tumor-bearing mice [18].

In clinical settings, APS in combination with chemotherapy increased the effectiveness of platinum-based chemotherapy [25] and further improved the quality of life for patients with advanced non-small cell lung cancer (NSCLC) [3].

Metformin is the most widely prescribed therapeutic agent for type 2 diabetes [26], and it is frequently researched for its role in tumor metabolism [27] and immune modulation [28,29,30].

Specifically, metformin has been reported to have effects on NSCLC according to in vitro and in vivo studies [31]. In laboratory studies, it has been found to decrease cell proliferation and induce apoptosis, and it is involved in oncogene signaling pathways [32,33]. The enhancement of chemotherapy, radiation, and glycolysis inhibitors was also observed [34,35,36]. Clinically, it was found to be a preventive treatment that decreased lung cancer risk retrospectively [37,38]. It has also been used in prospective clinical trials; however, the results on its effect in clinical trials remain inconsistent [39,40,41].

APS and metformin possess the characteristics of low toxicity and few side effects, as well as non-residue and non-tolerance properties, and therefore they are ideal for different adjuvant treatments [9]. In this study, we investigated the effect of APS on the lung adenocarcinoma A549 cell line, and metformin was used as a comparison model. Furthermore, the combination of these two drugs was also investigated in our study. 

## 2. Materials and Methods

### 2.1. Cell Viability Using APS, Metformin, and Combination Treatment

The A549 human lung adenocarcinoma cell line was obtained from the Bioresource Collection and Research Centre, Taiwan. Cells were cultured in an RPMI-1640 medium (Invitrogen Corp., Carlsbad, CA, USA) supplemented with 10% fetal bovine serum (FBS) at 37 °C in a 5% CO_2_ incubator.

The Astragalus polysaccharide (APS) was obtained from Carbosynth. Co., Limited, Compton, UK (product code: FA41820, from Mongolian Astragalus, using a low concentration of ethanol for precipitation and gel chromatography for purification; the approximate molecular weight was 301 kDa), while metformin HCl was obtained from Toronto Research Chemicals, CA, USA (product number: TRC-M258815, molecular weight: 165.62 g/mol).

A549 cells were grown to 60–70% confluence prior to treatment. Subsequently, the cells were treated with different concentrations of APS and metformin for the indicated time periods. The combination of APS with different concentrations of metformin was also used to treat A549 cell lines.

For further analysis, A549 cells were cultured in 96-well plates in a medium containing 10% FBS at a density of 1 × 10^4^ per well. Once the cells were attached, the medium was replaced with a fresh medium containing 10% FBS.

Cells were treated with the APS, metformin, and their combination for 24 h or 48 h incubation period. The absorbance was measured according to the manufacturer’s instructions using an XTT-based cell proliferation kit (Sartorius, Gottingen, Germany, cat. no. 20-300-1000). An EnSpire multimode plate reader (PerkinElmer Inc., Waltham, MA, USA) was used to quantify the XTT–formazan complex at an absorbance wavelength of 450 nm and a reference absorbance wavelength of 630 nm.

### 2.2. Apoptosis and Mitochondrial Membrane Potential Detection through Flow Cytometry Analysis

Following a previous study [42], A549 cells were seeded at a density of 2 × 10^5^ per well into a 6-well plate and cultured overnight prior to treatment. Subsequently, the medium was removed after different times and treatments, and the treated cells were collected. The medium was then removed after various times and treatments; the treated cells were then collected, and the supernatant was centrifuged and removed. The cells were resuspended and detected using the FITC-Annexin V Apoptosis Detection Kit (Biolegend, San Diego, CA, USA cat. no. 640914) and the Mitoscreen JC-1 Kit (BD Biosciences, cat. no. 551302) according to the manufacturer’s instructions. The BD FACSCanto II flow cytometer was used. Dot plots were used to present the analysis of apoptosis at different developmental stages.

### 2.3. Ferroptosis Effect Determination

#### 2.3.1. ROS Determination Assay

A549 cells were grown in six-well plates (2 × 10^5^/well) and treated with the above treatments for 24 or 48 h. The cells were washed and harvested. As per the manufacturer’s instructions, the ROS assay kit (Abcam, Cambridge, UK, ab113851) with propidium iodide (PI) staining was used to assess ROS activity. The procedure was performed according to the manufacturer’s instructions. In brief, cells were collected after the treatments, and a 2′,7′-dichlorofluorescin diacetate (DCFDA) was added to the cell suspension. It was then deacetylated to a non-fluorescent compound by cellular esterases, which was later oxidized by ROS to 2′,7′-dichlorofluorescein (DCF). DCF was highly fluorescent. It was detected with a BD FACSCanto II flow cytometer.

#### 2.3.2. Reduced Glutathione (GSH) and Malondialdehyde (MDA) Levels

The cells were cultured with different treatments for a certain duration and then harvested following the manufacturer’s instructions. A GSH/GSSG Ratio Detection Assay Kit (Abcam, Cambridge, UK, ab138881) was used to evaluate GSH levels, and MDA levels were assessed using a lipid peroxidation (MDA) assay kit (Abcam, Cambridge, UK, ab118970). The results were obtained using an EnSpire multimode plate reader (PerkinElmer Inc.).

#### 2.3.3. Protein Expression of Western Blotting Analysis and Protein Quantification

The treated A549 cells were harvested and extracted for Western blot analysis as previously mentioned [43]. Equal amounts of protein were fractionated on 10% SDS-PAGE. The fractionated proteins were transferred to polyvinylidene difluoride membranes. The membranes were blocked with a 5% *w/v* nonfat dry milk solution for a period of 60 min at room temperature. Membranes were incubated with primary antibodies overnight at 4 °C.

Primary antibodies were as follows: anti-PARP (anti-poly (ADP-ribose) polymerase, Cell Signaling, Danvers, MA, USA, #9542, 1:1000), anti-cleaved PARP (Cell Signaling #5625, 1:1000), anti-GPX4 (anti-glutathione peroxidase 4, Proteintech #14432-1-AP 1:1000), anti-β-actin antibody (Santa Cruz, Dallas, Texas, USA #47778, 1:1000), and anti-vinculin (Sigma-Aldrich, Burlington, MA USA #V9193, 1:12,000).

The primary and secondary antibodies were diluted with 1% nonfat dry milk in 0.1% TBST (Tris-buffered saline Tween-20). This was followed by washing with 1% TBST. They were then incubated with horseradish peroxidase-linked anti-mouse or anti-rabbit secondary antibodies (Cell Signaling, 1:5000) for one hour at room temperature. The protein signal was detected using the SuperSignal™ chemiluminescent substrate (Pierce, Rockford, IL, USA cat. no. 34087).

The Western blot band images were obtained using the G:BOX Chemi XX9 imaging system (Syngene). Protein level quantification was performed using ImageJ 1.54j. The band density of the untreated control group was used as the standard for the value ratio of the other groups.

### 2.4. Protein Level Quantification

The band images obtained by Western blotting were analyzed using AlphaEase^®^FC 3.0 software in accordance with the manufacturer’s instructions for protein level quantification. Following the selection of the band in each group and the subtraction of the background, the band densities were automatically calibrated. The density of the group without treatment was employed as the standard for the calculation of the ratio value of the other groups.

### 2.5. Statistical Analysis

Data are expressed as mean ± standard deviation (SD). Experiments were repeated at least three times. Differences between groups in XTT, apoptosis, and ferroptosis results were analyzed by analysis of variance (ANOVA). Tukey’s test was used as a post hoc test in ANOVA to test the significance of pairwise group comparisons. A *p* value ≤ 0.05 was considered statistically significant. All analyses were performed using GraphPad software 6 (Scientific, San Diego, CA, USA).

## 3. Results

### 3.1. Cell Viability under the Treatment of APS, Metformin Alone/APS in Combination with Metformin

**Figure 1:**
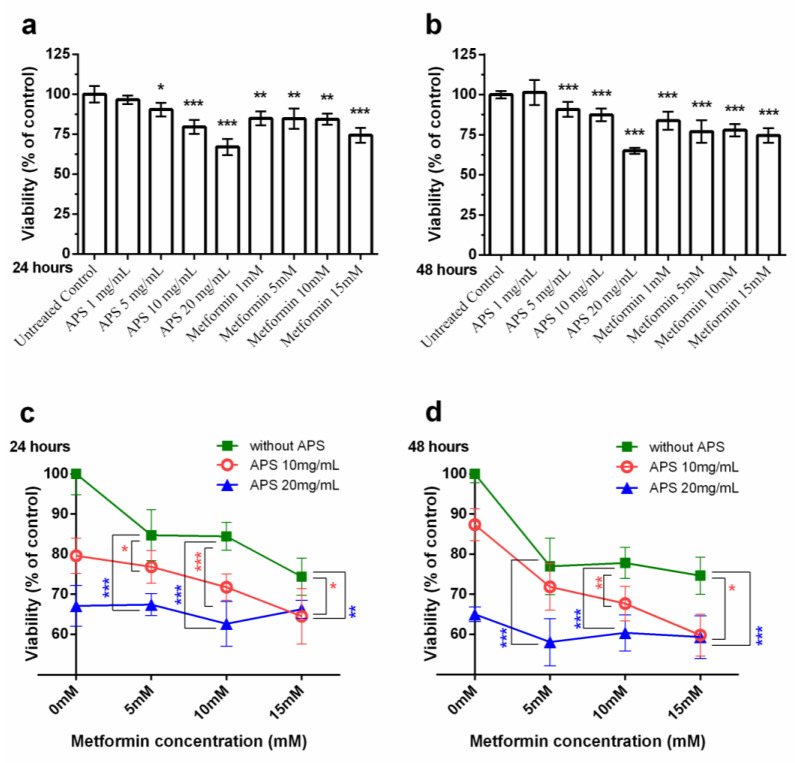
Synergistic effect of APS and metformin on A549 cell viability. A549 cell viability under the treatment of (**a**,**b**) APS/metformin alone for (**a**) 24 h and (**b**) for 48 h. (**c**,**d**) APS in combination with different concentrations of metformin after treatment of (**c**) 24 h and (**d**) 48 h. The combination group significantly decreased compared to using either APS or metformin alone. All the results are representative of at least three independent experiments. (Error bars = mean ± S.E.M. Asterisks mark samples significantly different from control group with (*) *p* < 0.05; (**) with *p* < 0.01; (***) with *p* < 0.001).

To investigate the effect of our medication on A549 cell growth, the cells were cultured with APS and metformin under different concentrations. As shown in Figure 1, a high concentration of APS significantly decreased the A549 cell growth. The inhibitory effect of metformin was also observed.

Metformin with a high concentration of APS was further used to treat A549 cells. However, the dose-dependent effect was more apparent with 10 mg/mL APS and not so apparent with 20 mg/mL APS.

### 3.2. Apoptotic Effects of APS Combined with Metformin

**Figure 2:**
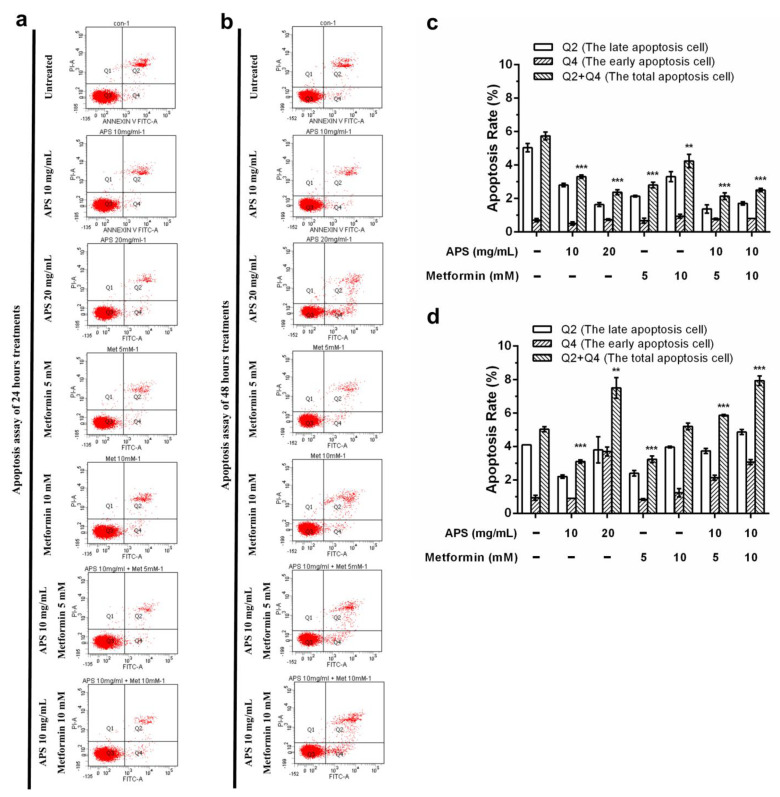
The apoptotic effect on A549 cells of APS, metformin detected by flow cytometry with annexin V-FITC/PI dual staining: (**a**,**c**) A549 cells treated for 24 h and (**b**,**d**) for 48 h. (Error bars = mean ± S.E.M. Asterisks mark samples significantly different from control group with (**) with *p* < 0.01; (***) with *p* < 0.001).

**Figure 3:**
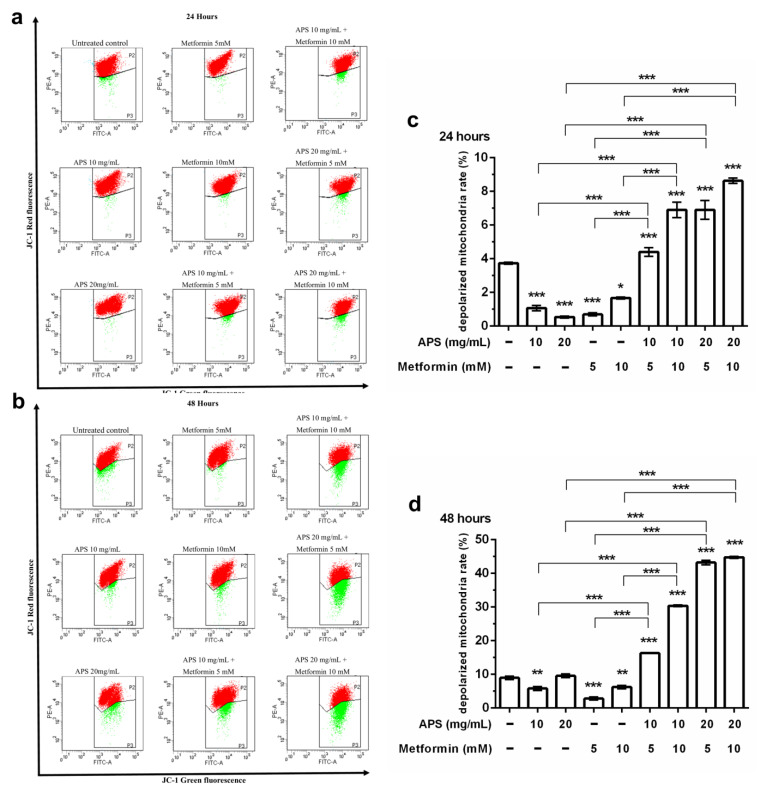
The mitochondrial depolarization stained with Mitoscreen JC-1 assay detected by flow cytometry. Dot plots showing depolarisation of mitochondria in treated A549 cells. The percentage of events in the upper gate (P2) and the percentage of events in the (P3) represent the population of treated A549 cells with normal and depolarised mitochondria, respectively. (**a**,**c**) A549 cells treated for 24 h and (**b**,**d**) for 48 h. (Error bars = mean ± S.E.M. Asterisks mark samples significantly different from control group with (*) *p* < 0.05; (**) with *p* < 0.01; (***) with *p* < 0.001).

**Figure 4:**
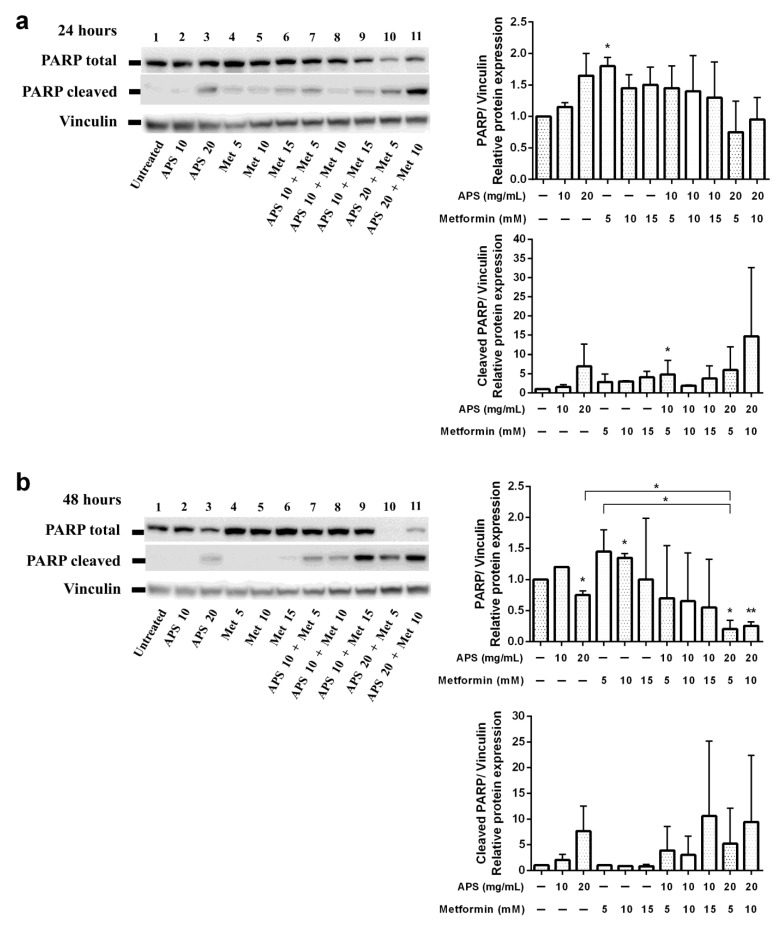
A549 were treated with indicated treatments for (**a**) 24 h and (**b**) 48 h and then harvested. The prepared cell protein was immunoblotted with polyclonal antibodies specific for PARP and cleaved PARP. Vinculin was used as an internal loading control. (Error bars = mean ± S.E.M. Asterisks mark samples significantly different from control group with (*) *p* < 0.05; (**) with *p* < 0.01).

Apoptosis assays were performed to verify the cellular death mechanism. The cells were treated with indicated treatments for different durations and then analyzed through flow cytometry with annexin V/PI double staining. From Figure 2d, it can be inferred that apoptotic cells significantly increased after 48 h treatment compared with the control group. Furthermore, as shown in Figure 3, mitochondrial depolarization was observed in all treatment groups after 24 h treatment. However, the effect was sustained until 48 h only in the combination treatment group. As shown in Figure 4, the upregulation of cleaved PARP was seen in a high concentration of APS and metformin after 24 h treatment. The sustained effect was also observed in the combination group after 48 h.

### 3.3. The Effects of APS, Metformin, and Their Combination on A549 Cell Ferroptosis

In addition to apoptotic cell death, ferroptosis may play a significant role in several diseases, including malignant tumors [43,44,45,46]. Thus, we investigated the mechanism behind the activity of APS and metformin in lung cancer A549 cells to verify this assumption.

**Figure 5:**
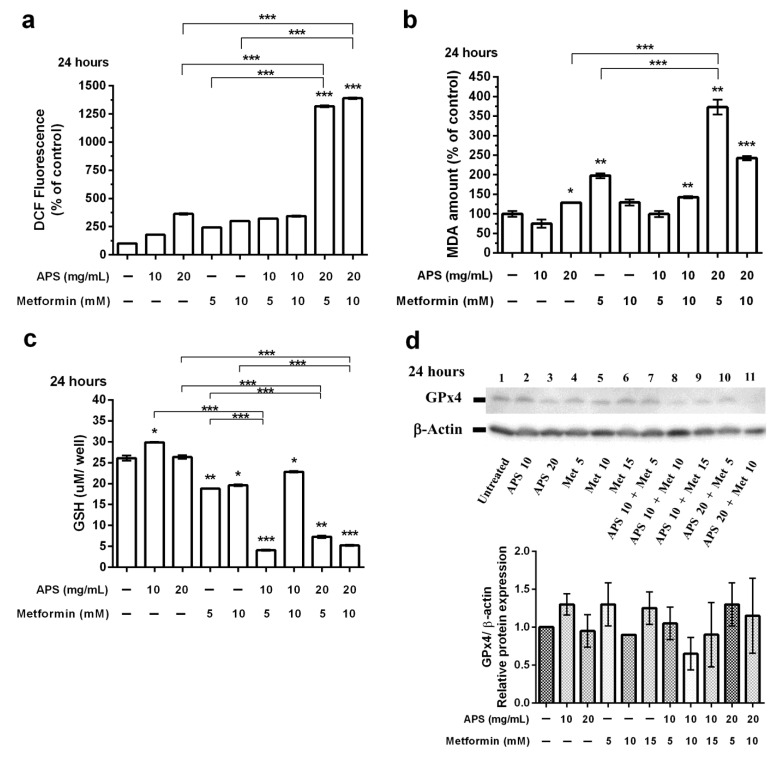
Effect of APS, metformin, and their combination on ferroptosis of A549 cells in vitro. The A549 cells were treated with either APS, metformin, or their combination under different concentrations for 24 h and then collected for the following test: (**a**) ROS assay; (**b**) MAD assay; (**c**) GSH levels; (**d**) Western blot for GPx4 protein. B-actin was used as the internal control. (Error bars = mean ± S.E.M. Asterisks mark samples significantly different from control group with (*) *p* < 0.05; (**) with *p* < 0.01; (***) with *p* < 0.001).

The ROS levels were measured after 24 h (Figure 5a) of the indicated treatment. ROS accumulation increased significantly with the combination treatment, namely 20 mg/mL of APS combined with 5 and 10 mM of metformin. Additionally, MDA levels increased with the treatment of APS, metformin, and their combination after 24 h (Figure 5b). However, the dose-dependent effect was not observed.

GSH and GSSG form a crucial cellular antioxidant system that maintains a reducing environment to decrease ROS levels. GPX4 plays a vital role in regulating ferroptosis [42]. As shown in Figure 5c, the reduction in GSH levels was significant in metformin and combination groups. Additionally, GPx4 inhibition (Figure 5d) was also observed after the 24 h treatment.

## 4. Discussion

In this study, the effect of the natural compound APS derived from the TCM herb *Astragalus membranaceus* interfering with lung adenocarcinoma A549 cells was investigated. A synergistic effect of APS and metformin was observed in the results.

The XTT assay results (Figure 1) revealed that A549 cells were inhibited by a high concentration of APS (10 mg/mL, 20 mg/mL) and metformin (15 mM). The additive effect was seen when APS was combined with metformin. The combination of 10 mg/mL of APS with 5 mM of metformin significantly decreased the A549 cell growth. In the XTT assay, considering the 24 and 48 h treatments, the effect of both medications was similar. Additionally, the dose-dependent effect was not significant with higher additional metformin.

As shown in Figure 2, the apoptotic effect of A549 cells was not apparent under annexin V/PI dual staining. Nevertheless, the elevation of the apoptotic effect of the combination treatment and time-dependent effect could still be observed. Mitochondrial depolarization (Figure 3) was significantly increased when 24 h; however, only the combination groups revealed the sustained effect after 48 h treatment. As shown in Figure 4, the PARP protein Western blot also revealed similar results.

For ferroptosis, the effects of 24 h treatment were more apparent than those of 48 h treatment. Figure 5 reveals that the effects of combination groups varied under different concentrations, which may imply that the mechanism and the effect of APS and metformin may be different for A549 cells.

APS has been widely researched in treating various cancers. Most studies have found that APS has a potential role in the immunomodulation of cancer cells. For example, macrophage activation was observed in using APS for treating breast cancer cells [21]. For hepatocellular carcinoma, increased cell apoptosis was seen with concentration-dependent APS treatment [22]. Additionally, for lung cancer, the reduction and inhibition of transcriptional activities of p65 mRNA and NF-κB transduction on NSCLC cell lines were reported [47]. 

Metformin is often researched for its involvement in the metabolic reprogramming of tumor cells, commonly referred to as ‘aerobic glycolysis’ [48,49]. For instance, metformin significantly downregulated lung cancer cell glycolysis and enhanced the treatment of tyrosine kinase inhibitor afatinib [50]. It should be noted that inhibiting tumor metabolism with metformin may require high doses, which can lead to lactic acidosis [51]. Combination therapy with other antitumor adjuvants shows promise in reducing the required dosage of metformin.

Nevertheless, the characteristics of APS and metformin are alike. Both drugs can attenuate immunity, regulate glucose metabolism, and have low toxicity and few side effects. They have already been widely used in clinical practice. The additive effect of these two medications may imply their involvement in the immunity modulation and metabolism of non-small cell lung cancer A549 cells. To our knowledge, this is the first report on the potential of using a combination of APS and metformin as adjuvant therapy. Further research on more cancer types and in vivo research may be needed to investigate the exact effect of this combination on cancer treatment.

## Data Availability

The raw data supporting the conclusions of this article will be made available by the authors upon request.
